# Plasma β-Endorphin and Neuropeptide Y as Candidate Biomarkers for Predicting Obstructive Sleep Apnea Syndrome: A Preliminary Study

**DOI:** 10.1155/carj/4316574

**Published:** 2025-09-15

**Authors:** Meng-Lin Li, Yi Yang, Qian-Yun Huang, Jian-Yong Liu

**Affiliations:** ^1^Department of Otolaryngology, Head and Neck Surgery, Zhangjiagang Hospital Affiliated to Soochow University, The First People's Hospital of Zhangjiagang City, Suzhou 215600, Jiangsu, China; ^2^Soochow University, Suzhou 215600, Jiangsu, China

**Keywords:** β-endorphin, apnea–hypopnea index, body mass index, neuropeptides, neuropeptide Y, obstructive sleep apnea, predictive value

## Abstract

**Background and Objective:** Given the involvement of neuropeptides in the pathophysiology of obstructive sleep apnea syndrome (OSAS), this study investigated the associations between plasma levels of β-endorphin (β-EP) and neuropeptide Y (NPY) and OSAS severity and evaluated their potential as predictive biomarkers.

**Methods:** A total of 48 snoring patients undergoing polysomnography (PSG) were categorized into non-OSAS, mild, moderate, and severe OSAS groups (*n* = 12 per group) based on the apnea–hypopnea index (AHI). Plasma levels of β-EP and NPY were measured using ELISA. Statistical analyses included one-way ANOVA, Spearman's correlation, and receiver operating characteristic (ROC) curve analysis to assess predictive performance.

**Results:** Plasma β-EP levels exhibited significant elevation in moderate (*p*=0.003) and severe OSAS groups (*p*=0.032) compared to the non-OSAS group. Notably, NPY levels demonstrated marked differences across all OSAS severity groups (*p* < 0.01), with significantly higher concentrations observed in mild, moderate, and severe OSAS patients versus non-OSAS controls (*p* < 0.01). A progressive increase in NPY levels was observed with advancing OSAS severity, accompanied by statistically significant intergroup differences (*p* < 0.01). Correlation analyses revealed strong positive associations between NPY levels and both BMI (*p* < 0.0001) and AHI (*p* < 0.0001). In contrast, β-EP correlated positively with AHI (*p* < 0.0001) but not with BMI (*p*=0.0931). ROC curve analysis identified β-EP (cutoff: 9.405 ng/L) as a moderate predictor of OSAS (AUC = 0.7986, *p* < 0.01; sensitivity: 72.22%, specificity: 83.33%). Strikingly, NPY (cutoff: 19.29 ng/L) exhibited perfect discriminative capacity (AUC = 1, *p* < 0.0001; sensitivity: 97.22%, specificity: 100%).

**Conclusions:** Plasma β-EP and NPY levels are associated with OSAS severity and may serve as potential biomarkers. However, further validation in larger cohorts is needed to confirm their clinical utility.

## 1. Introduction

Obstructive sleep apnea syndrome (OSAS) is a condition in which the upper airway repeatedly collapses during sleep to cause snoring, frequent apneas, repeated hypoxemia, sleep structure disorders, superficial breathing, and hyperpnea at nighttime, thus resulting in daytime sleepiness, arrhythmia, hypertension, cerebrovascular accidents, and multiorgan damage, which can be life-threatening sometimes [1–3]. Growing evidence highlights the critical role of biomarkers in understanding OSAS pathophysiology and clinical management. Recent studies have identified diverse biomarkers associated with OSAS severity and complications, including inflammatory markers such as YKL-40 [4], metabolic indicators such as Lp-PLA2 [5], and regulatory noncoding RNAs such as circulating miRNA panels [6]. These findings underscore the multifactorial nature of OSAS and the potential of biomarker-based approaches to improve diagnosis and risk stratification.

β-endorphin (β-EP), an endogenous opioid neuropeptide, directly modulates respiratory control by suppressing medullary chemoreceptor sensitivity during hypoxia [7] while simultaneously participating in stress responses and metabolic homeostasis [8]. In contrast, neuropeptide Y (NPY) contributes to obesity-related OSAS through its involvement in appetite stimulation, fat accumulation via hypothalamic pathways [3, 9] and amplification of hypoxic stress via peptidylglycine α-amidating monooxygenase (PAM)–mediated upregulation during intermittent hypoxia [10].

Critically, although prior studies have independently linked these biomarkers to OSAS [3, 11], none have simultaneously evaluated their combined correlation with both apnea–hypopnea index (AHI) and BMI across the full OSAS severity spectrum, a gap this study addresses. Our novel dual-biomarker approach not only clarifies their distinct contributions (respiratory suppression vs. metabolic dysregulation) but may also improve diagnostic precision over single-parameter models, particularly in differentiating metabolic-driven vs. anatomical OSAS subtypes. Emerging evidence demonstrates the biomarker potential of β-EP and NPY across diverse clinical contexts, with β-EP effectively predicting IVF outcomes in reproductive disorders [12] and NPY showing significant associations with neuropsychiatric conditions including suicidal behavior [13]. These findings underscore their broad pathophysiological relevance while highlighting the need for disease-specific validation, particularly in OSAS, where their combined predictive utility remains unexplored.

Therefore, this study aimed to elucidate the relationships between β-EP, NPY, and OSAS severity and determine their utility as biomarkers for predicting OSAS. Clinically, our findings could aid in the development of novel diagnostic tools and provide insights into the pathophysiological mechanisms underlying OSAS. By identifying biomarkers with high predictive value, we aim to contribute to improved screening, timely diagnosis, and management of OSAS, particularly in high-risk populations.

## 2. Methods

### 2.1. Subjects

Forty-eight snoring patients who attended the Sleep Medicine Center, Department of Otolaryngology, Zhangjiagang Hospital, Soochow University, from October 2017 to December 2018, were collected for polysomnography (PSG). According to their AHI, the patients were categorized into mild OSAS group (5 ≤ AHI < 20), moderate OSAS group (20 ≤ AHI < 40), severe OSAS group (AHI ≥ 40), and non-OSAS group (AHI < 5) [14].

The inclusion criteria were: (1) 20–50 years of age; (2) having quitted smoking and alcohol for at least 2 weeks before PSG and venous blood sampling; (3) drinking no coffee, tea, energy drinks and other beverages that may affect sleep quality during 24 h before PSG; (4) other factors that may influence PSG results. The exclusion criteria were: (1) combined with serious systemic diseases like cerebrovascular accident, diabetes and coronary heart disease; (2) combined with deviation of the nasal septum, chronic hypertrophic rhinitis and other diseases that may affect nasal ventilation; (3) pregnant women. A total of 48 eligible subjects were recruited into this study, with 12 cases in each group. Participants were required to maintain their regular dietary habits and refrain from new medications (except essential treatments) during the study period. While detailed psychological assessments were not conducted, subjects with known psychiatric disorders were excluded.

This study was approved by the Ethics Committee of the Affiliated Zhangjiagang Hospital of Soochow University (Approval number: 201709016). Informed consent document was obtained from each subject.

### 2.2. Observation Indicators

The primary observation indicators included plasma levels of β-EP and NPY, as measured by an enzyme-linked immunosorbent assay (ELISA). Baseline patient information including age, gender, and BMI was recorded. In addition, the AHI and lowest arterial oxygen saturation (LaSO_2_) indicators were recorded through PSG. Correlation analyses were performed to evaluate the relationships between β-EP, NPY, AHI, and BMI. Receiver operating characteristic (ROC) curves were generated to determine the predictive value of β-EP and NPY for OSAS diagnosis, including sensitivity, specificity, and optimal cutoff values.

### 2.3. Samples and Methods

Venous blood samples (5 mL) were collected from each subject at 8:00 a.m. after overnight fasting for 12 h and placed in anticoagulant tubes containing EDTA and then gently mixed and transferred to tubes containing aprotinin. After gentle mixing, blood samples were centrifuged at 3000 rpm/min for 10 min (centrifugal radius, 70 mm) and were preserved at −70°C. Plasma levels of β-EP and NPY were measured by commercial ELISA kits (R&D System, USA) following related recommendations. ELISA assays were performed by technicians blinded to the clinical status and group assignments of participants.

### 2.4. Statistical Analysis

Statistical analyses were performed using SPSS 22.0 (IBM SPSS, USA). Enumeration data were evaluated using the chi-square test. Measurement data were expressed as mean ± SD and subjected to the normal distribution test (Shapiro–Wilk test) and the homogeneity of variance test. Normally distributed data with homogeneity between groups were analyzed using the Student's *t*-test, and those among three or more groups using the one-way ANOVA. The correlation between the two factors was assessed by Spearman's correlation test. The ROC of GraphPad Prism 6 (GraphPad Software, USA) was used to obtain the best cutoff values of β-EP and NPY for predicting OSAS. The area under the curve (AUC) was used to determine the value of β-EP and NPY in the diagnosis of OSAS. *p* < 0.05 was considered as statistically significant.

## 3. Results

### 3.1. Baseline Characteristics of Participants

The baseline characteristics of participants, including sex, age, and LaSO_2_, are summarized in Table 1. There were no significant differences in sex distribution (*p*=0.938) or age (*p*=0.707) among the groups. However, LaSO_2_ significantly decreased with increasing OSAS severity (*p* < 0.001), with the severe OSAS group showing notably lower values compared to the non-OSAS, mild, and moderate OSAS groups.

### 3.2. BMI and AHI Comparisons Between Groups

Shapiro–Wilk test results verified that the BMIs of the four groups were normally distributed, with evident homogeneity of variance (Table 2). There was a significant difference in the BMI among the four groups, as shown by the one-way ANOVA (*p*=0.003). Notably, a significant difference in the BMI was observed between the non-OSAS group and the severe OSAS group (*p*=0.002) and between the mild OSAS group and the severe OSAS group (*p*=0.031) but not between the other groups (*p* > 0.05). The BMI also showed a significant difference in the rank sum test (*p*=0.022). After Bonferroni correction, a significant difference in BMI was observed between the severe OSAS group and the non-OSAS group (*p*=0.003), while other comparisons did not reach statistical significance.

Shapiro–Wilk tests showed that the AHI values of the four groups were non-normally distributed, without obvious homogeneity of variance even after correction (*p*=0.002, Table 2). Thereafter, the AHI values were compared by the Kruskal–Wallis *H* test, and a significant difference was obtained (*p* < 0.001). After Bonferroni correction, a significant difference in AHI was observed between the non-OSAS group and the moderate OSAS group (*p* < 0.001), between the non-OSAS group and the severe OSAS group (*p* < 0.001), as well as between the mild OSAS group and the severe OSAS group (*p* < 0.001).

### 3.3. β-EP and NPY Comparisons Between Groups

Shapiro–Wilk test results verified that the plasma levels of β-EP in the four groups were normally distributed with obvious homogeneity of variance (Table 3). There was a significant difference in the plasma level of β-EP among the four groups, as shown by the one-way ANOVA (*p*=0.003). Notably, the plasma level of β-EP was significantly higher in the moderate OSAS group and the severe OSAS group than in the non-OSAS group (*p*=0.003and *p*=0.032, respectively), while no significant difference was found between the other groups (*p* > 0.05).

The plasma level of NPY was normally distributed with obvious homogeneity of variance (Table 3). The level showed a significant difference among the four groups (*p* < 0.001). NPY was significantly higher in the mild, moderate, and severe OSAS groups than in the non-OSAS group (*p* < 0.01). Moreover, the level increased with the severity of OSAS (*p* < 0.01).

### 3.4. Correlation Analysis

**Figure 1:**
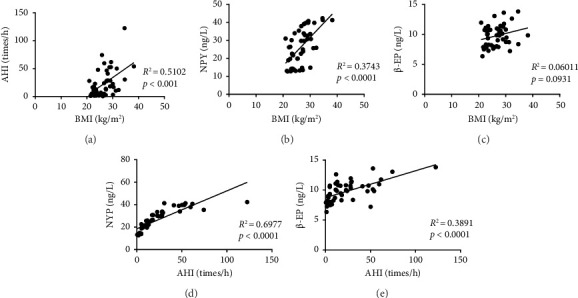
Correlation analyses of BMI, AHI, and the plasma levels of β-EP and NPY. (a) BMI and AHI. (b) BMI and NPY. (c) BMI and β-EP. (d) AHI and NPY. (e) AHI and β-EP. Abbreviations*:* BMI: body mass index; AHI: apnea–hypopnea index; β-EP: β-endorphin; NPY: neuropeptide Y.

For the data that were normally distributed, the Pearson's correlation test was performed, otherwise Spearman's correlation test was adapted. We analyzed all patients, and the results showed that BMI was positively correlated with AHI (*R*^2^ = 0.5102, *p* < 0.001, Figure 1(a)), and it was also positively correlated with plasma NPY (*R*^2^ = 0.3743, *p* < 0.0001, Figure 1(b)) but not with plasma β-EP (*R*^2^ = 0.06011, *p* > 0.05, Figure 1(c)). Meanwhile, AHI was not only positively correlated with plasma NPY (*R*^2^ = 0.6977, *p* < 0.0001, Figure 1(d)) but also with plasma β-EP (*R*^2^ = 0.3894, *p* < 0.0001, Figure 1(e)).

### 3.5. Values of β-EP and NPY in Predicting OSAS

**Figure 2:**
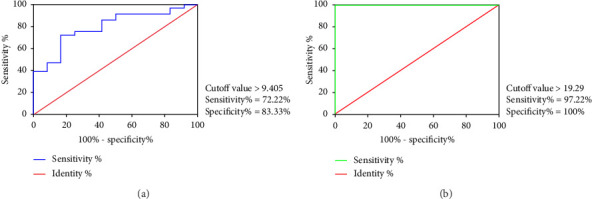
ROC for the plasma levels of β-EP and NPY in predicting OSAS. (a) β-EP. (b) NPY. Each panel shows the AUC, cutoff value, sensitivity, and specificity values. Abbreviations: ROC: receiver operating characteristic curve; AUC: area under the curve; β-EP: β-endorphin; NPY: neuropeptide Y; OSAS: obstructive sleep apnea syndrome.

We used GraphPad Prism 6 software to perform ROC. For β-EP, a cutoff value of 9.405 ng/L yielded an AUC of 0.7986 (95% CI: 0.6628–0.9344), with a sensitivity of 72.22% and a specificity of 83.33% for predicting OSAS (*p* < 0.01, Figure 2(a)). For NPY, the optimal cutoff value of 19.29 ng/L achieved an AUC of 1, with a sensitivity of 97.22% and a specificity of 100% in predicting OSAS (*p* < 0.0001, Figure 2(b)). These results indicated that NPY demonstrated near-perfect discriminative capacity in this preliminary cohort, warranting cautious interpretation, given the sample size.

## 4. Discussion

Obese people usually have short, thick necks and narrow upper airways, predisposing them to OSAS symptoms, such as airway obstruction, snoring, and decreased blood oxygen saturation during sleep. A long-term OSAS may cause cardiovascular diseases (e.g., arrhythmia, coronary heart disease, and hypertension) and metabolic diseases (e.g., hyperlipidemia and diabetes). It is reported that people with a higher BMI have higher risks of cardiovascular and metabolic diseases [15], and the severity of snoring in obese patients is closely linked to cardiovascular remodeling [10]. In the present study, we identified a positive correlation between BMI and AHI in recruited snorers. Moreover, more overweight and obese subjects were found in the OSAS group than in the non-OSAS group. It is speculated that obese and overweight people are prone to developing OSAS, and more importantly, obesity may further aggravate clinical symptoms and signs of OSAS. Therefore, declining BMI is conductive to reducing the risk of OSAS, hypopnea, and hypoxemia, and potential cardiovascular and metabolic diseases. Obese people with OSAS are recommended to perform enough exercise to lose weight and to lower their BMI.

The pathogenesis and potential risk factors of OSAS remain unclear. Current evidences demonstrate that β-EP, NPY, vasoactive intestinal peptide, apolipoprotein B-100, fibronectin, and ceruloplasmin may be involved in the development of OSAS [11, 16]. Among them, β-EP and NPY can stimulate appetite and participate in energy metabolism, probably leading to obesity, which is considered to increase the risk of OSAS [3, 8, 11]. Our findings identified that plasma levels of NPY had a significant positive correlation with BMI, and β-EP had a positive correlation trend with BMI but not significant. However, both β-EP and NPY were positively correlated with AHI, supporting their links to disease severity. Although AHI derived from PSG remains the diagnostic standard, its single-night, in-lab nature limits continuous tracking of respiratory variability. Recent dual-pathway deep-learning–based respiratory monitoring achieves high-precision, real-time rhythm tracking, and could complement biomarker testing to enhance dynamic AHI assessment in OSAS [17]. Collectively, these findings support β-EP/NPY as severity-linked biomarkers while underscoring the need for longitudinal and mechanistic studies to clarify causality and determine whether these neuropeptide changes are adaptive or maladaptive in OSAS.

Furthermore, we observed a significant decrease in the LaSO_2_ with increasing OSAS severity. LaSO_2_ reflects the degree of nocturnal hypoxemia, which is a hallmark of OSAS and has been associated with increased oxidative stress, inflammation, and sympathetic activation [7, 18]. The significantly lower LaSO_2_ observed in the severe OSAS group highlights the extent of hypoxic burden in these patients and may help explain the compensatory rise in β-EP and NPY levels.

NPY, which is widely distributed in the central and peripheral nervous systems, regulates hormone secretion, feeding behavior, and cardiovascular functions via its receptors [19]. NPY also stimulates one's appetite and reduces fat utilization and energy consumption, leading to fat accumulation and even obesity, which will further aggravate OSAS [3]. An animal experiment has shown that intermittent hypoxia can effectively activate the PAM in the rat brainstem, while PAM can catalyze the production of NPY to increase the NPY level in the blood [20]. Furthermore, NPY also affects sleep regulation and circadian rhythms and is involved in various nervous system processes and neuropsychiatric disorders, such as anxiety disorders [21]. Previous data have revealed the increased incidence of depression and anxiety in patients with OSAS [22, 23], suggesting that NPY may also have a role in mental and psychological disorders in patients with OSAS, which requires further exploration. Our results showed that NPY had a high predictive value for OSAS, whose sensitivity was 97.22% and the specificity was 100%, thus making it a good candidate biomarker to predict OSAS.

As a neuropeptide with opioid bioactivity secreted by the pituitary gland, β-EP in the form of neurohormones regulates many complex physiological functions [8]. Increased level of β-EP in the blood suppresses the sensitivity of the medulla oblongata of the respiratory center, aortic sinus, and aortic body chemoreceptors, forming a vicious cycle of hypoxia and β-EP increase; therefore, the pathophysiological changes of OSAS occur [24]. In addition, an animal experiment verified the role of β-EP in accelerating the progression and instability of atherosclerotic plaques [25]. Our study revealed that the plasma level of β-EP in both moderate and severe OSAS groups was significantly higher than that in the non-OSAS group, indicating the role of β-EP in the development of OSAS. Further ROC analysis showed that β-EP had moderate predictive value for OSAS, which made it possible to test serum β-EP to predict OSAS in clinical practice.

Several limitations in this preliminary study should be noted. First, the small sample size limits statistical power and increases the risk of overfitting, particularly regarding the ROC analysis of NPY, which yielded perfect discrimination (AUC = 1.0). While promising, this finding should be interpreted with caution. Larger cohorts with internal validation (e.g., bootstrapping) are needed to confirm this finding. Second, although we controlled for major comorbidities through strict inclusion/exclusion criteria, we did not systematically assess other potential confounding variables such as psychological status, specific dietary components, medication use, or smoking history. These factors may influence neuropeptide levels and should be evaluated using standardized instruments in future research. Third, we focused primarily on BMI as a clinical correlate, whereas other important OSAS-related parameters, such as ODI, minimum oxygen saturation, sleep architecture, and metabolic markers, were not included in the analysis. Incorporating these physiological measures could improve the depth and interpretability of biomarker associations. In addition, the study investigated only two biomarkers (β-EP and NPY), while numerous other bioactive molecules have been implicated in OSAS pathogenesis. Future work should expand the biomarker panel to provide a more comprehensive view of the neuroendocrine and inflammatory mechanisms involved. Moreover, although β-EP and NPY are biologically plausible candidates, their clinical translation is limited by assay cost, lack of standardized testing protocols, and variability across populations; addressing these barriers through methodological standardization, affordable rapid assays, and multicenter validation is essential before their integration into routine OSAS screening. Finally, multivariate modeling was not performed in this study. Our analysis was limited to univariate associations to explore initial trends, but we acknowledge that adjusting for confounders such as age, sex, and BMI is essential for establishing independent predictive value. Future studies should incorporate multivariable regression models and validation cohorts to enhance clinical relevance and generalizability.

In conclusion, plasma levels of β-EP and NPY were significantly associated with the severity of OSAS, suggesting that the plasma β-EP and NPY may serve as potential biomarkers for predicting OSAS severity. Nevertheless, these findings need to be validated by more multicenter or large-sample clinical studies.

## Figures and Tables

**Table 1:** Baseline characteristics of participants.

Group	Non-OSAS	Mild OSAS	Moderate OSAS	Severe OSAS	*p*
*n*	12	12	12	12	
Sex					0.938
Male	10	10	9	10	
Female	2	2	3	2	
Age (years)	46.25 ± 15.357	47.83 ± 10.161	48.92 ± 10.370	43.25 ± 13.322	0.707
LaSO_2_ (%)	92 ± 2.17	89.58 ± 2.87	85.73 ± 7.59	74.75 ± 12.41^abc^	< 0.001

Abbreviations: LaSO2, lowest arterial oxygen saturation; OSAS, obstructive sleep apnea syndrome.

^a^ *p* < 0.05 vs. non-OSAS.

^b^ *p* < 0.05 vs. mild OSAS.

^c^ *p* < 0.05 vs. moderate OSAS.

**Table 2:** BMI and AHI values' comparisons between groups.

Group	*n*	BMI^∗^	BMI classification^∗∗^			AHI^#^
			Normal	Overweight	Obesity	
Non-OSAS	12	24.60 ± 2.57	7 (58.3%)	2 (16.7%)	3 (25.0%)	2.71 ± 1.51
Mild OSAS	12	25.89 ± 3.27	5 (41.7%)	3 (25.0%)	4 (33.3%)	9.75 ± 3.20
Moderate OSAS	12	26.45 ± 3.30	4 (33.3%)	1 (8.3%)	7 (58.3%)	23.39 ± 4.93
Severe OSAS	12	29.93 ± 4.10	0 (0.0%)	3 (25.0%)	9 (75.0%)	57.85 ± 23.06

Abbreviations: AHI, apnea–hypopnea index; BMI, body mass index; OSAS, obstructive sleep apnea syndrome.

^∗^One-way ANOVA, *p*=0.003.

^∗∗^Rank sum test, *p*=0.022.

^#^Kruskal–Wallis *H* test, *p* < 0.001.

**Table 3:** Comparisons of plasma levels of β-EP and NPY between groups.

Group	*n*	β-EP (ng/L)^∗^	NPY (ng/L)^∗∗^
Non-OSAS	12	8.47 ± 1.28	13.63 ± 0.72
Mild OSAS	12	9.48 ± 1.71	22.28 ± 2.41
Moderate OSAS	12	10.32 ± 1.03	30.60 ± 2.30
Severe OSAS	12	10.82 ± 2.00	39.20 ± 2.45

Abbreviations: β-EP, β-endorphin; NPY, neuropeptide Y; OSAS, obstructive sleep apnea syndrome.

^∗^One-way ANOVA, *p*=0.003.

^∗∗^One-way ANOVA, *p* < 0.001.

## Data Availability

All data generated or analyzed are included in this article. Further inquiries can be directed to the corresponding author.

## Nomenclature

AHI: Apnea–hypopnea index
AUC: Area under the curve
β-EP: β-endorphin
BMI: Body mass index
NPY: Neuropeptide Y
OSAS: Obstructive sleep apnea syndrome
PAM: Peptidylglycine α-amidating monooxygenase
PSG: Polysomnography
ROC: Receiver operating characteristic curve
